# Evaluation of the efficacy of humic acids to counteract the toxic effects of aflatoxin B1 in turkey poults

**DOI:** 10.3389/fvets.2023.1276754

**Published:** 2023-10-10

**Authors:** Jesús Adonai Maguey-González, María de Jesús Nava-Ramírez, Sergio Gómez-Rosales, María de Lourdes Ángeles, Bruno Solís-Cruz, Daniel Hernández-Patlán, Rubén Merino-Guzmán, Xochitl Hernandez-Velasco, Juan Omar Hernández-Ramírez, Ileana Loeza, Roberto Senas-Cuesta, Juan D. Latorre, Alma Vázquez-Durán, Xiangwei Du, Abraham Méndez-Albores, Billy M. Hargis, Guillermo Téllez-Isaías

**Affiliations:** ^1^Posgrado en Ciencias de la Producción y de la Salud Animal, Universidad Nacional Autónoma de México (UNAM), Unidad de Posgrado, Ciudad Universitaria, Ciudad de México, México; ^2^Department of Poultry Science, University of Arkansas, Fayetteville, AR, United States; ^3^Unidad de Investigación Multidisciplinaria L14 (Alimentos, Micotoxinas, y Micotoxicosis), Facultad de Estudios Superiores (FES) Cuautitlán, UNAM, Cuautitlán Izcalli, Estado de México, México; ^4^Centro Nacional de Investigación Disciplinaria en Fisiología y Mejoramiento Animal (CENID-INIFAP), Km1 Carretera a Colon Ajuchitlán, Querétaro, México; ^5^Laboratorio 5: LEDEFAR, Unidad de Investigación Multidisciplinaria, FES Cuautitlán, UNAM, Cuautitlán Izcalli, Estado de México, México; ^6^División de Ingeniería en Nanotecnología, Universidad Politécnica del Valle de México, Tultitlan, México; ^7^Departamento de Medicina y Zootecnia de Aves, Facultad de Medicina Veterinaria y Zootecnia, UNAM, Ciudad de México, México; ^8^Veterinary Medical Diagnostic Laboratory, Department of Biomedical Sciences, University of Missouri, Columbia, MO, United States

**Keywords:** aflatoxin B1, humic acids, adsorbents, turkey poults, performance parameters

## Abstract

This study aims to evaluate the efficacy of humic acid (HA) from worm compost as an adsorbent for aflatoxin B_1_ (AFB_1_) in turkey poults. The experiment involved the inclusion of 0.25% (w/w) HA in the diet of turkey poults consuming aflatoxin-contaminated feed (250 ng AFB_1_/g). A total of 350 1-day-old female Nicholas-700 turkey poults were randomly allocated to five equal groups: negative control (basal diet); positive control (basal diet + 250 ng AFB_1_/g; HA (basal diet + 0.25% HA); HA + AFB_1_ (basal diet + HA + 250 ng AFB_1_/g); and zeolite + AFB_1_ (basal diet + 0.25% zeolite + 250 ng AFB_1_/g). Each group had seven replicates of 10 poults (*n* = 70). The impact of HA addition was evaluated in terms of performance parameters, relative organ weights, liver histological lesions, and serum biochemical and hematological constituents. In general, the addition of HA improved body weight (BW), body weight gain (BWG), and feed conversion rate (FCR). Furthermore, HA effectively mitigated the toxic effects caused by AFB_1_ in the majority of the analyzed variables. The results indicated that HA effectively counteracted the AFB_1_-induced toxic effects in turkey poults. Based on these findings, it can be concluded that HA is capable of removing AFB_1_ from the contaminated diet.

## 1. Introduction

Aflatoxin B_1_ (AFB_1_) is a highly toxic mycotoxin, primarily produced by *Aspergillus flavus* and *Aspergillus parasiticus*, and it poses a significant concern in poultry farming, especially for turkey poults (1, 2). Thus, extensive research has been conducted to investigate the harmful effects of AFB_1_ in animals, including their carcinogenic, mutagenic, teratogenic, and growth-inhibitory properties (3, 4). Contamination with aflatoxins leads to reduced feed quality and animal performance due to poor nutrient conversion and various issues, such as reproductive abnormalities (5). Aflatoxicosis in poultry manifests as listlessness, decreased appetite, reduced growth rate, inefficient feed utilization, decreased egg production, and increased mortality. Additionally, aflatoxin contamination in feed can impair both humoral and cellular immune responses, rendering birds more susceptible to environmental and infectious agents (4).

Previous studies have extensively reported the presence of potential aflatoxin-producing strains and AFB_1_ molecules in commercial poultry feeds. As a result, there is an urgent need for practical and cost-effective methods to decontaminate aflatoxin-contaminated feed on a large scale (6). Several physical, chemical, and biological techniques have been employed to decontaminate agricultural commodities with good results, but their implementation has been limited by uncertainty about their *in vivo* effectiveness (7). One practical approach is the utilization of non-nutritive adsorbent materials, which can effectively bind mycotoxins and inhibit their absorption in the gastrointestinal tract (8), thereby reducing their toxic impact on poultry and minimizing the carryover of these fungal metabolites into poultry products such as meat-end eggs (9). However, not all adsorbents exhibit significant effectiveness, and some of them have been found to hinder nutrient utilization. An optimal adsorbent material should exhibit a high affinity for aflatoxins, facilitating the formation of stable complexes with minimal risk of dissociation. Furthermore, it should possess a substantial binding capacity to prevent saturation (10).

Humic substances (HS) are widely distributed and can be found in areas undergoing decomposition or sedimentation, such as soils (11). Within the HS, three distinct components are distinguished based on their solubility: fulvic acids (FA), humic acids (HA), and humins (12). These substances have demonstrated a strong ability to bind various compounds, including heavy metals, herbicides, mutagens, aromatic compounds, minerals, and bacteria (13). Recent studies have shown that incorporating HS into poultry diets promotes growth by enhancing energy digestibility and nutrient retention (14, 15). Although the specific mechanisms behind this growth promotion are not fully understood, the inclusion of HA in the chicken diet leads to elevated intestinal viscosity and improved intestinal integrity (16, 17). In a recent study, HA derived from vermicompost demonstrated remarkable efficacy in adsorbing AFB_1_ in an *in vitro* model that simulates the digestive tract of birds (18). Therefore, this study aimed to evaluate the effects of HA on performance parameters, relative organ weight, liver histological lesions, and serum biochemical and hematological analyses in AFB_1_-exposed turkey poults.

## 2. Materials and methods

### 2.1. Animal sources, diets, and experimental design

A total of 350 1-day-old female Nicholas-700 turkey poults (Aviagen Inc., AR, USA) were raised in pens for 28 days. Poults were collectively weighed (10 birds/pen) and randomly allocated to one of the five groups: negative control (basal diet); positive control (basal diet + 250 ng AFB_1_/g; HA (basal diet + 0.25% HA); HA + AFB_1_ (basal diet + HA + 250 ng AFB_1_/g); and zeolite + AFB_1_ (basal diet + 0.25% zeolite + 250 ng AFB_1_/g). Each group had seven replicates of 10 poults (*n* = 70). A maize-soybean-based turkey poult diet was formulated to approximate the nutritional requirements recommended by the National Research Council (17) and adjusted to the breeder's recommendations (Table 1). AFB_1_, HA, and zeolite were added to the diet and mixed thoroughly to the specified level. Poults had *ad libitum* access to water and feed during the experiment. All animal handling procedures complied with the Institutional Animal Care and Use Committee (IACUC) at the University of Arkansas, Fayetteville (protocol no. 22020).

**Table 1:** Composition of the experimental diet.

**Ingredient**	**Turkey starter diet (%)**
Corn	43.33
Soybean meal	42.24
Animal protein concentrate^*^	7.50
Poultry fat	3.40
Limestone	0.66
Dicalcium phosphate	1.52
Salt	0.24
DL-methionine	0.38
L-lysine HCl	0.42
L-threonine	0.11
Vitamin/mineral premix^**^	0.15
Choline chloride (60%)	0.05
**Calculated composition**	(%)
Crude protein	28
AME (kcal/kg)	3,020
Total Ca	1.49
Available phosphorus	0.76
Dig Total sulfur amino acids (TSAA)	1.06
Dig lysine	1.64
Dig threonine	0.96
Dig isoleucine	1.01
Dig valine	1.12
Dig tryptophan	0.28
Dig arginine	1.75

^*^Composition: crude protein, 57%; crude fat, 8.5%; calcium, 7.94%; phosphorus, 3.59%; sodium, 0.49%; potassium, 0.38%; chloride, 0.73%; cysteine, 1%; methionine, 0.71%; lysine, 3.13%; histidine, 0.91%; tryptophan, 0.34%; threonine, 1.97%; arginine, 3.78%; isoleucine, 1.88%; leucine, 3.71%; phenylalanine, 2.09%; valine, 2.77% (H.J. Baker's ProPlus 57%).

^**^Supplied per kg of feed by vitamin and mineral premix (0.15%): Vitamin A, 13,227 IU; vitamin D_3_, 6,613 ICU; vitamin E, 66 IU; calcium, 51 mg; manganese, 124.5 mg; zinc, 124.5 mg; copper, 7.5 mg; iodine, 2.1 mg; selenium, 0.3 mg.

### 2.2. Aflatoxin

AFB_1_ was provided by Dr. Xiangwei Du, Veterinary Medical Diagnostic Laboratory, University of Missouri, Columbia, MO, USA. AFB_1_ was produced through the fermentation of rice (19) using an *Aspergillus parasiticus* strain. The aflatoxin content was determined using high-performance liquid chromatography with a fluorescence detection method, utilizing a Romer derivatization unit (Romer Labs Inc., Washington, MO) (20). The composition consisted of 86.82% AFB_1_, 8.22% AFG_1_, 4.55% AFB_2_, and 0.37% AFG_2_. Diets were analyzed to verify the actual AFB_1_ concentration.

### 2.3. Humic acids

The method for extracting and isolating HA from worm compost was conducted following the protocol outlined by (21) with minor modifications. The extraction of HA involved stirring the compost in a sodium hydroxide solution (1M) at a ratio of 1:4 (compost: alkali) for 2 h. The resulting suspension was allowed to stand at room temperature for 24 h and subsequently filtered using a Whatman grade 40 filter paper. The filtrate was centrifuged at 3,500 × *g* for 15 min, and the supernatant was carefully decanted. To precipitate the HA, the supernatant containing HA was acidified using hydrochloric acid (10%, v/v) with continuous agitation until reaching pH 2. HA was separated from fulvic acid through centrifugation at 3,500 × *g* for 15 min. The resulting precipitate (HA) was adjusted to pH 10 with 1M NaOH and subsequently dried at 60°C. Finally, the dried powder was finely ground using a Thomas Willey grinder and sieved through a 0.25-mm mesh.

### 2.4. Zeolite

A non-commercial zeolitic material was employed as a reference. The main elements in the zeolite were Si (40.93%), C (31.99%), O (14.13%), and Al (7.46%), with smaller amounts of Na (0.25%), K (1.80%), Mg (1.12%), Ca (1.45%), and Fe (0.87%). Characterization details are fully described by Maguey-González et al. (18).

### 2.5. Performance parameters

Each pen replicates served as an individual experimental unit. Poults were collectively weighed each week to obtain body weight (BW) and body weight gain (BWG). The feed intake (FI) and feed conversion ratio (FCR) were determined weekly.

### 2.6. Relative organ weights

All birds were euthanized on day 28 by CO_2_ inhalation. The intestine, liver, spleen, and bursa of Fabricius were removed and weighed (21 birds per treatment). The relative organ weight ratio was calculated as follows:


$$\begin{array}{l} \text{Relative weight }=\frac{\left(\text{Organ weight}\right)}{\left(\text{Body weight}\right)}\text{x }100 \end{array}$$

### 2.7. Histological evaluation of liver tissue

The liver (three birds from each replicate) was removed on day 28. Hepatocellular degeneration and lymphoid and heterophilic infiltration were evaluated. Liver sections were fixed in neutral buffered formalin (10%) until processed. A transversal section of the middle part of the left hepatic lobule was prepared routinely, dehydrated in increasing alcohol concentrations, and embedded in paraffin. A 5-μm thick tissue sample was cut, stained with hematoxylin and eosin, and mounted with coverslips for the histological analysis.

Hepatocellular degeneration was scored as follows: Score: 0 (normal or absence of cellular swelling), 1 (mild vacuolar degeneration/fat deposition), 2 (moderate vacuolar degeneration/fat deposition), and 3 (severe vacuolar degeneration/fat deposition). Additionally, congestion of vessels, bile duct hyperplasia, and fibrosis were scored. The score was obtained by evaluating five fields with a magnification of 20 × per section of tissue. The scores were considered per treatment.

Quantification of inflammatory cells (lymphocytes) was obtained using an adapted methodology previously described (22). In a general field from the upper left end of the tissue cut, with a magnification of 53, an area of 3.4 mm^2^ was evaluated. The lesion score was assigned by counting the number of cell layers from the center of the cell cluster or the space of the perivascular area toward the perimeter of the cluster where the greatest number of cell layers were present. The lesion score was as follows: Score: 0 normal/minimal inflammatory infiltrate (1–11 cell layers/cluster), 1 mild inflammatory infiltrate (12–24 cell layers/cluster), 2 moderate inflammatory infiltrate (25–50 cell layers/cluster), and 3 severe inflammatory infiltrate (51–100 cell layers/cluster).

### 2.8. Serum biochemical analysis

At the end of the experiment, 21 birds from each treatment were randomly selected, and blood was collected from their femoral veins. Blood was centrifuged at 1,118 × *g* at 4°C for 15 min. The serums were taken and preserved at −20°C until submitted for biochemical analysis. Serum concentrations of total protein, alanine aminotransferase (ALT), alkaline phosphatase (ALP), aspartate aminotransferase (AST), creatine kinase (CK), glutamate dehydrogenase (GLDH), creatinine, blood urea nitrogen (BUN), glucose, sodium, chloride, calcium, phosphorus, uric acid, and CO_2_ were determined using a Corning clinical chemistry analyzer (Chiron Corporation, San Jose, CA).

### 2.9. Hematological analysis

Fourteen birds from each treatment (2 birds/replicate) were randomly selected and bled from the femoral vein. Whole blood was collected in EDTA blood tubes and subjected to automated hematology analysis (Cell-Dyn; Abbott Diagnostics, Abbott Park, IL). Data acquired included the concentration of white cells, hematocrit (%), heterophils, lymphocytes, monocytes, basophils, and heterophils/lymphocytes ratio.

### 2.10. Statistical analysis

Data from performance, relative organ weights, serum biochemicals, and hematological analysis were subjected to ANOVA as a complete randomized design, using the general linear model procedure of SAS (23). The Duncan multiple range tests at P < 0.05 determined significant differences among means.

Data from lesion scores of liver histopathological analysis are expressed as median (mode; variance), and differences among median values of the groups were analyzed using the Mann-Whitney *U*-test with a level of significance set at *P* < 0.05.

## 3. Results

Table 2 summarizes differences in BW, BWG, FI, and FCR in turkeys consuming a corn-soybean-based diet contaminated with AFB_1_ (250 ng AFB_1_/g) added with HA. The differences in BW started decreasing compared to the positive control group (221.92 g) and both HA treatments (249.49 and 241.09 g for HA and HA+AFB_1_, respectively) at day 14 (*P* < 0.0001). At day 21, the BW of both HA treatments (428.35 and 390.10 g for HA and HA+AFB_1_, respectively) was higher than the AFB_1_ treatments (348.78 g) (*P* < 0.0001). Similarly, at day 28, the BW of both HA treatments (637.47 and 571.95 g for HA and HA+AFB_1_, respectively) was higher than the AFB_1_ treatments (428.14 g) (*P* < 0.0001). Differences in BWG were observed from the second week (7–14 days). The BWG of both HA treatments was higher than the AFB_1_ treatments (*P* < 0.0001). Furthermore, the BWG of HA treatment was 178.85 g in the third week (14–21 d) (*P* < 0.0001) and 209.12 g in the fourth week (21–28 days) (*P* < 0.0001), followed by HA+AFB_1_ treatment (149.01 and 181.85 g, respectively) compared to positive control treatment (126.86 and 85.14 g, respectively). When comparing from 0–28 days, the BWG of both HA treatments remained significantly higher (581.13 and 515.25 g for HA and HA+AFB_1_, respectively) compared to the positive control treatment (371.76 g) (*P* < 0.0001). Furthermore, during the last week (21–28 days), the FI was significantly higher in both HA treatments (288.00 and 266.80 g for HA and HA+AFB_1_, respectively) when compared to the positive control treatment (141.75 g) (*P* < 0.0001). With respect to the FI from 0–28 days, it was significantly higher in both HA treatments (764.04 and 706.76 g for HA and HA+AFB_1_, respectively) compared to the positive control treatment (141.75 g) (*P* < 0.0001). Moreover, the FCR was consistently higher in the positive control group than in all the other treatments throughout the entire duration of the experiment. Finally, the FCR from 0–28 days was significantly higher in the negative control (1.56), positive control (1.52), and ZEO+AFB_1_ (1.51) treatments compared to both HA treatments (1.31 and 1.37 for HA and HA+AFB_1_, respectively) (*P* < 0.0001), denoting a better FC in the treatments added with HA.

**Table 2:** Evaluation of body weight (BW), body weight gain (BWG), feed intake (FI), and feed conversion ratio (FCR) in turkeys consuming a corn-soybean-based diet contaminated with aflatoxin B1 (250ppb) supplemented with humic acids.

**Parameter**	**Negative control**	**Positive control**	**HA**	**HA + AFB1**	**ZEO + AFB1**	**SEM**	***P*-value**
**BW, g**							
Day 0	56.78	56.11	56.34	56.70	56.54	0.93	0.70
Day 7	115.11	119.74	122.25	122.49	116.3	6.20	0.11
Day 14	224.11^b^	221.92^b^	249.49^a^	241.09^a^	217.72^b^	11.46	< 0.0001
Day 21	365.03^bc^	348.78^cd^	428.35^a^	390.10^b^	329.52^d^	25.06	< 0.0001
Day 28	483.54^c^	428.14^d^	637.47^a^	571.95^b^	440.78^d^	33.94	< 0.0001
**BWG, g**							
Days 0–7	58.33	63.62	65.91	65.79	59.81	6.18	0.09
Days 7–14	108.99^b^	102.17^b^	127.24^a^	118.59^a^	101.36^b^	8.05	< 0.0001
Days 14–21	140.91^b^	126.86^bc^	178.85^a^	149.01^b^	111.79^c^	19.88	< 0.0001
Days 21–28	118.51^c^	85.14^d^	209.12^a^	181.85^b^	111.26^c^	22.85	< 0.0001
Days 0–28	426.76^c^	371.76^d^	581.13^a^	515.25^b^	384.23^d^	33.78	< 0.0001
**FI, g**							
Days 0–7	88.54	92.45	93.06	85.55	85.09	7.24	0.15
Days 7–14	184.92^a^	161.30^b^	172.81^ab^	166.16^b^	161.15^b^	13.85	0.01
Days 14–21	239.63^a^	198.52^b^	246.77^a^	207.63^b^	219.84^ab^	25.49	0.007
Days 21–28	178.96^b^	141.75^b^	288.00^a^	266.80^a^	172.15^b^	39.01	< 0.0001
Days 0–28	668.44^b^	567.76^c^	764.04^a^	706.76^ab^	581.84^c^	55.28	< 0.0001
**FCR**							
Days 0–7	1.52^a^	1.46^a^	1.41^ab^	1.30^b^	1.4^ab^	0.10	0.01
Days 7–14	1.72^a^	1.57^ab^	1.36^c^	1.40^bc^	1.59^ab^	0.16	0.002
Days 14–21	1.55^a^	1.40^ab^	1.17^c^	1.27^bc^	1.47^a^	0.16	0.001
Days 21–28	1.46^b^	1.67^a^	1.37^c^	1.46^bc^	1.55^b^	0.08	< 0.0001
Days 0–28	1.56^a^	1.52^a^	1.31^b^	1.37^b^	1.51^a^	0.05	< 0.0001

^abc^Means with non-matching superscripts within rows indicates a significant difference at *P* < 0.05. Seven replicates/group (*n* = 10 poults/replicate).

Table 3 shows the relative weight of the liver, spleen, bursa of Fabricius, and intestine in poults consuming diets contaminated with AFB_1_ and HA. The relative weight of the liver was lower (*P* < 0.0001) in the positive control group (3.72 g) compared to the negative control group (2.89 g) and the HA (3.35 g) poults, while it was similar to the zeolite+AFB_1_ group (2.98 g) and higher than the HA+AFB_1_ (2.56) poults. The AFB_1_ inclusion in the positive control (0.15 g) caused an increase in the relative weight of the spleen (*P* < 0.006) compared to the negative control (0.12), and it showed similar values compared to the rest of the treatments. The negative control (0.17 g) poults showed a similar relative weight of the bursa of Fabricius compared to the positive control (0.19 g) and the zeolite+AFB_1_ (0.20 g) treatments, while it was lower compared to the HA (0.22 g) and HA+AFB_1_ (0.22 g) poults. The relative weight of the intestine of the positive control (8.25 g) was similar to that of the negative control (8.66 g) and the zeolite+AFB_1_ (8.07 g) treatment, which in turn were higher (*P* < 0.0001) than that of the HA (6.96 g) and HA+AFB_1_ (6.42 g) treatments. No significant differences were observed in the bursa/spleen ratio among treatments.

**Table 3:** The relative weight of liver, spleen, bursa of Fabricius, and intestine in turkeys consuming a corn-soybean-based diet contaminated with 250 ppb of aflatoxin B1 during 28 days, supplemented with humic acids.

	**Relative weight (g)**						
	**Negative control**	**Positive control**	**HA**	**HA** + **AFB1**	**ZEO** + **AFB1**	**SEM**	***P*** **-value**
Liver	3.72^a^	2.89^c^	3.35^b^	2.56^d^	2.98^c^	0.43	< 0.0001
Spleen	0.12^c^	0.15^ab^	0.14^abc^	0.13^bc^	0.16^a^	0.38	0.006
Bursa of Fabricius	0.17^c^	0.19^bc^	0.22^a^	0.22^a^	0.20^ab^	0.04	0.0006
Intestine	8.66^a^	8.25^a^	6.96^b^	6.42^b^	8.07^a^	1.12	< 0.0001
Bursa/spleen ratio	147.01	133.74	160.74	175.98	135.63	52.95	0.06

^abc^Means with non-matching superscripts within rows indicates a significant difference at *P* < 0.05. Seven replicates/group (*n* = 3 poults/replicate).

Table 4 presents the blood cell counts of poults consuming diets contaminated with AFB_1_ and HA. No significant differences were observed in the white cells, hematocrit, heterophils, lymphocytes, basophils, or heterophils/lymphocyte ratio. The monocyte counts in the negative control (1,500/μL), positive control (2,423/μL), and HA+AFB_1_ (2,208/μL) poults were similar, and the counts were higher (*P* < 0.0001) between the HA (6,127/μL) and zeolite+AFB_1_ (5,648/μL) treatments.

**Table 4:** Blood count cells in turkeys consuming a corn-soybean-based diet contaminated with 250ppb of aflatoxin B1 during 28 days, supplemented with humic acids.

	**Negative Control**	**Positive Control**	**HA**	**HA+AFB1**	**ZEO+AFB1**	**SEM**	***P*-value**
White cells (10^3^/μL)	15.87	20.58	20.70	20.58	21.80	8.72	0.67
Hematocrit (%)	40.75	36.60	38.70	41.10	42.10	6.44	0.34
Heterophils (10^3^/μL)	6,421	5,169	4,715	8,631	7,588	4,463	0.27
Lymphocytes (10^3^/μL)	6,966	6,749	8,726	8,961	7,677	2,881	0.33
Monocytes (10^3^/μL)	1,500^b^	2,423^b^	6,127^a^	2,208^b^	5,648^a^	2,031	< 0.0001
Basophils (10^3^/μL)	961	650	1111	780	820	629	0.54
Heterophils/Lymphocytes	0.98	0.73	0.77	0.98	1.07	0.76	0.79

^abc^Means with non-matching superscripts within rows indicates a significant difference at *P* < 0.05. Seven replicates/group (*n* = 2 poults/replicate).

Table 5 presents the serum biochemical parameters in poults consuming diets contaminated with AFB_1_ supplemented with HA. The inclusion of AFB_1_ the positive control induced a significant decrease in serum levels of total protein (*P* < 0.0001), AST (*P* < 0.001), GLDH (*P* < 0.02), creatinine (*P* < 0.0001), BUN (*P* < 0.0009), glucose (*P* < 0.002), Ca (*P* < 0.0001), and P (*P* < 0.0002) compared to the negative control treatment. In poults receiving the diet with added HA, there was a partial restoration of total protein, creatinine, and Ca compared to the positive control, and there was a lower concentration of ALT, ALP, AST, BUN, glucose, P, and uric acid, but higher Na, Cl, and CO_2_ concentrations compared to the negative control. All the biochemical parameters were similar between HA+AFB_1_ and the positive control poults, with the exception of CO_2_, which was higher in the former group. In zeolite+AFB_1_ poults, the total protein, ALT, creatinine, and BUN were higher, and Ca concentrations were lower than the positive control.

**Table 5:** Serum biochemical parameters in turkeys consuming a corn-soybean-based diet contaminated with 250ppb of aflatoxin B1 during 28 days, supplemented with humic acids.

	**Negative control**	**Positive control**	**HA**	**HA + AFB1**	**ZEO + AFB1**	**SEM**	***P*-value**
Total Protein (g/dL)	3.82^a^	1.68^d^	3.10^b^	1.62^d^	2.02^c^	0.16	< 0.0001
ALT (U/L)	4.80^ab^	3.00^bc^	2.20^c^	2.60^c^	6.20^a^	1.54	0.002
ALP(U/L)	2,569.40^a^	2,561.40^a^	1,970.8^b^	2,633.60^a^	2,321.60^a^	241.72	0.001
AST(U/L)	302.60^a^	256.00^b^	235.60^b^	258.80^b^	301.00^a^	25.45	0.001
CK (U/L)	6,644	3,832	2,140	2,491	7,878	234.03	0.06
GLDH (U/L)	11.00^a^	8.00^bc^	9.60^abc^	7.20^c^	10.20^ab^	1.90	0.02
Creatinine (mg/dL)	0.20^a^	0.11^c^	0.17^ab^	0.09^c^	0.15^b^	0.02	< 0.0001
BUN (mg/dL)	4.60^a^	3.60^b^	2.80^b^	3.20^b^	4.60^a^	0.67	0.0009
Glucose (mg/dL)	583.00^a^	405.00^b^	453.00^b^	414.20^b^	430.80^b^	66.14	0.002
Sodium (mEq/L)	140.80^b^	141.20^b^	148.40^a^	142.40^b^	139.80^b^	3.69	0.01
Chloride (mEq/L)	105.40^b^	107.60^b^	111.40^a^	107.60^b^	105.60^b^	2.72	0.01
Calcium (mg/dL)	9.04^a^	5.76^c^	6.90^b^	5.32^cd^	4.74^d^	0.69	< 0.0001
Phosphorus (mg/dL)	12.06^a^	9.12^b^	8.50^b^	8.12^b^	9.60^b^	1.15	0.0002
Uric acid (mg/dL)	10.80^a^	9.74^ab^	6.56^c^	8.70^b^	10.24^ab^	1.36	0.0007
CO_2_ (mEq/L)	11.70^c^	15.36^b^	18.44^a^	17.18^a^	13.70^b^	1.32	< 0.0001

^abcd^Means with non-matching superscripts within rows indicates significant difference at *P* < 0.05. Seven replicates/group (*n* = 3 poults/replicate).

**Figure 1:**
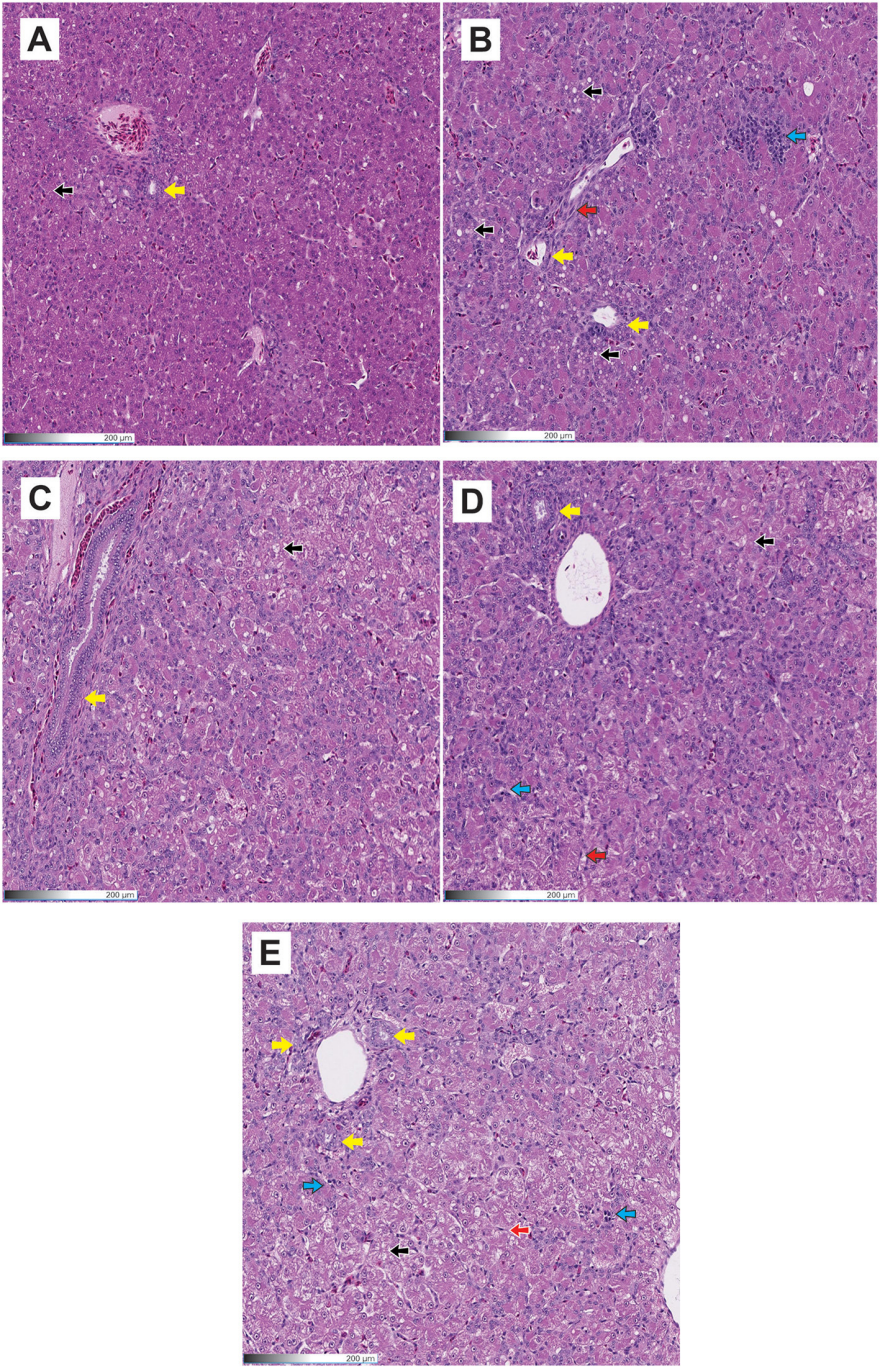
Histological images were taken using a 20X objective on H & E stained tissue sections of the liver. **(A)** Negative control; **(B)** Positive control; **(C)** HA; **(D)** HA+AFB_1_; **(E)** zeolite+AFB_1_. Black arrows show vacuolar degeneration; Blue arrows show inflammation; Yellow arrows show bile duct hyperplasia; Red arrows show fibrosis.

Table 6 summarizes the hepatocellular degeneration responses in poults consuming diets contaminated with AFB_1_ and supplemented with HA. The positive control treatment, combined with AFB_1_, showed significant increases (*P* < 0.05) in cellular degeneration and fibrosis compared to the negative control. In HA poults, lower (*P* < 0.05) vessel congestion was found compared to the positive and negative control groups, and lower bile duct hyperplasia was observed compared to the positive control group. The findings were corroborated by histopathology studies, which showed that the liver of turkeys from the AFB_1_ group had substantial cellular degeneration and fat deposition (Figures 1A–E). In HA+AFB_1_ poults, the cell degeneration was higher compared to the negative control, while the rest of the variable responses were similar compared to the positive and negative controls and the HA poults.

**Table 6:** Hepatocellular degeneration in turkeys consuming a corn-soybean-based diet contaminated with 250ppb of aflatoxin B1 during 28 days, supplemented with humic acids.

	**Negative control**	**Positive control**	**HA**	**HA + AFB1**	**ZEO + AFB1**
Cell degeneration	1.00 (1.00–1.89)^b^	2.50 (2.00–3.00)^a^	1.50 (1.00–2.89)^ab^	2.50 (2.00–3.00)^a^	3.00 (2.00–3.00)^a^
Vacuolar degeneration	1.50 (0.11–2.89)^a^	1.00 (1.00–3.00)^a^	1.50 (0.11–2.89)^a^	1.50 (0.00–3.00)^a^	1.00 (0.11–2.00)^a^
Congestion of vessels	2.00 (1.00–2.00)^a^	2.00 (1.00–2.00)^a^	1.00 (1.00–1.00)^b^	1.50 (0.11–2.89)^ab^	1.00 (0.11–2.00)^ab^
Lymphocytic infiltration	1.00 (0.00–1.89)^a^	1.50 (1.00–2.89)^a^	1.00 (0.00–1.89)^a^	2.00 (1.00–3.00)^a^	1.00 (0.11–1.89)^a^
Bile duct hyperplasia	0.00 (0.00–1.89)^ab^	1.50 (1.00–2.00)^a^	0.50 (0.00–1.00)^b^	1.00 (0.00–1.89)^ab^	0.50 (0.00–2.00)^ab^
Fibrosis	0.00 (0.00–0.89)^b^	1.00 (1.00–1.00)^a^	0.00 (0.00–1.00)^ab^	0.50 (0.00–1.00)^ab^	0.00 (0.00–0.00)^c^

Data are expressed as median (LCI-UCI at 95%). ^a, b^Superscripts within rows indicate a significant difference (*P* < 0.05), according to Mood's median test with its respective chi-square test; *n* = 12. LCI, lower confidence intervals; UCI, upper confidence intervals.

Regarding the zeolite+AFB_1_-fed poults, the cell degeneration was higher than the negative control. Meanwhile, vacuolar degeneration, congestion vessels, lymphocytic infiltration, and bile duct hyperplasia were similar to positive, negative, and HA poults. Conversely, fibrosis exhibited the lowest occurrence compared to all the other treatments.

## 4. Discussion

The exposure of turkeys to AFB_1_ negatively impacts productivity, causing significant economic losses and health problems in the poultry industry because of its toxicity (24). In an earlier study, a significant decrease in BWG and FCR was observed in poults fed diets with quantities equal to or higher than 400 ppb from 2 to 7 weeks of age (25). In poults fed naturally contaminated whole corn kernels with AFB_1_ from 1 to 21 days of age, reduced BWG was observed (26). Reduced FI and BW were also seen in poults from 1 to 21 days of age fed diets added with 100 ppb of aflatoxins and from 1 to 42 days of age when diets contained 200 ppb of aflatoxins (27). In poults fed 100 ppb aflatoxin, reduced BWG was observed, and at higher inclusions of aflatoxins, reduced FI and higher mortalities were found (28). Other recent publications have reported reduced BWG and increased FCR in poult-fed aflatoxin-contaminated feeds (29, 30). The reduced BW and BWG and increased FCR in poults fed the positive control diet in the present study agree with previous findings, which show that the growth performance of poults is negatively affected by the consumption of AFB_1_.

Conversely, poults fed with HA showed increased BW, BWG, and FI, and reduced FCR, which is consistent with previous findings on broiler chickens supplemented with HS derived from worm composts (15). It is worth noting that HA-fed poults outperformed the negative control ones in BW, BWG, FI, and FCR by 31.8, 36.2, 14.3, and 16.0%, respectively. Interestingly, HA+AFB_1_-fed poults also improved the BW, BWG, FI, and FCR by 18.3, 20.7, 5.7, and 12.2%, respectively, compared to those of the negative control group. These results indicate that HA-fed turkeys not only restored the productive parameters that were adversely affected by AFB_1_ in the positive control group but also improved the productivity of the negative control group. To the best of our knowledge, this is the first study where HA supplementation is reported to improve the productivity of unchallenged and AFB_1_-challenged poults.

HS has demonstrated a strong ability to bind various compounds, including heavy metals, herbicides, mutagens, aromatic compounds, minerals, and bacteria (13). In a recent study, HA derived from vermicompost was characterized and found to be highly effective in adsorbing AFB_1_ in an *in vitro* model simulating a chicken's digestive tract (18). Due to its highly hydrophobic surfaces and a wide range of negatively charged functional groups (31), interactions between HA and AFB_1_ can occur through various mechanisms. Tan (9) has identified seven potential binding mechanisms for HA with gaseous, liquid, and solid components, which include (i) physical forces, (ii) chemical forces, (iii) hydrogen bonds, (iv) hydrophobic interactions, (v) electrostatic interactions, (vi) coordination reactions, and (vii) ligand exchange. Among these mechanisms, electrostatic interactions and hydrogen bonding play crucial roles in the HA-AFB1 interaction.

Additionally, other interactions, such as π- π stacking and hydrophobic interactions, can also be considered. Although this phenomenon is still being studied, it seems that aromatic structures and functional groups such as OH and COOH are also involved. Further details about the hypothetical mechanism by which HA binds the AFB_1_ molecule are found in a previous study by our research group (18).

Another important result was that zeolite+AFB_1_ poults had BW, BWG, and FI similar to those in the positive control but lower than the negative control group. The FCR was similar among the positive control, negative control, and zeolite+AFB_1_-fed poults. These results indicate that the protective effect of zeolite against AFB_1_ was overlooked in the present study. Zeolite is known to bind AFB_1_, preventing its absorption into the body, and has been shown to be partially protective against high levels of AFB_1_ (32, 33). In the literature, no studies looking at the protective effects of zeolite against AFB_1_ on the performance of turkey poults were found. Nonetheless, it was recently demonstrated that supplementation with zeolite in the diets of female and male turkey poults had a positive effect on growth performance compared to the control group (34). This topic deserves further investigation.

In terms of relative liver weight, the negative control treatment had the highest, followed by the treatments with HA, HA+AFB_1_, zeolite+AFB_1_, and the positive control. Similar to these findings, in poults fed AFB_1_-contaminated corn, a reduction in liver weight was reported, while in poults fed uncontaminated corn, increased liver weight was observed (26). Reduced liver weight in poults fed aflatoxin-contaminated feeds was also reported compared to a control group fed non-contaminated feed (30). Rauber et al. (27) conducted a study on poults with different aflatoxin levels (20–1,000 ppb). The liver weight of control poults and those fed diets with aflatoxin levels between 20 and 500 ppb showed no significant difference, with the exception of the group fed 50 ppb, which displayed a lower liver weight. However, poults that were fed diets containing 1,000 ppb of aflatoxins demonstrated an increase in liver weight (27). Furthermore, enlargement of the liver was reported in poults fed AFB_1_-contaminated feeds (35). These discrepancies regarding the effect of AFB_1_ feeding on the liver of poults need further research.

A larger spleen was reported in poults fed aflatoxin-contaminated corn (26) and rations (35), while spleens of similar weight between poults fed aflatoxin-contaminated and non-contaminated feeds were reported (30). In this research, the larger spleen was found in zeolite+AFB_1_ and positive control poults and the smallest in the negative control group. These results agree with research in which larger spleens were seen in poults fed aflatoxin-contaminated feeds. Concerning the bursa of Fabricius, both HA treatments demonstrated higher weights, and the positive control showed lower weights. Different from these findings, significant atrophy and degenerative changes were observed in the bursa in a study involving 7-day-old female turkey poults challenged with 0.2 mg AFB_1_/kg; the changes included degeneration, necrosis, and depletion of lymphoid cells (36). The higher weight of the bursa of Fabricius in poults fed aflatoxin-contaminated corn was also reported (26), while in other reports, a lower bursa weight was recorded in poults fed aflatoxin-contaminated feed (30, 35). In HA and HA+AFB_1_-fed poults, the weight of the intestine was lower; this was surprising since, in other animal species, it has been reported that HS increases the size of the intestine, the viscosity in the lumen, the mucosal surface area, and the number of goblet cells, which leads to increased intestinal weight (17, 37).

No significant differences were found in the number of cells, hematocrit, heterophils, lymphocytes, basophils, or hetero/lymph ratio. Significant differences were observed only in monocyte counts in the HA treatment. Monocytes are derived from the bone marrow and released into the bloodstream. They have a brief circulation in the blood before migrating into the tissues and differentiating into tissue macrophages. Macrophages play critical roles in various physiological functions, including inflammation, wound healing, immune regulation, and metabolic homeostasis. Therefore, a decrease in the number of peripheral blood monocytes in birds fed contaminated diets suggests a potential reduction of synthesis in the bone marrow or increased clearance from the bloodstream (38). Our findings corroborate the results of a previous study conducted by Bujnák et al. (39), which also reported no significant changes in red and white blood cell parameters with the addition of HS on blood variables in finishing pigs.

In the AFB_1_ treatment group, a notable decrease in serum levels of total protein, AST, CK, GLDH, creatinine, BUN, glucose, Ca, and P was observed when compared to the negative control treatment. These findings indicate that AFB_1_ exposure significantly impacts various serum markers, suggesting potential alterations in liver function and metabolic processes. The inclusion of AFB_1_ in the feed had detrimental effects on serum protein, cholesterol, glucose, and uric acid levels, while the activity of AST and ALT enzymes increased in the turkey poults and poultry chickens (40). Furthermore, the activities of γ-glutamyl transferase, lactate dehydrogenase, and aspartate aminotransferase were found to be elevated in poultry exposed to AFB_1_ (1, 35).

HA exhibited positive effects on certain serum enzyme activities and helped alleviate the serum biochemical changes associated with aflatoxin toxicity (41). Moreover, there were reductions observed in the levels of serum albumin, total protein, uric acid, and cholesterol with HA supplementation (42). The protection of HS against liver injury was also evaluated in several studies in which hepatotoxicity was induced using lipopolysaccharide, carbon tetrachloride, and ethanol in rats, reporting reduced AST, ALT, and ALP serum concentrations (43, 44).

Compared to the negative control, the positive control exhibited a significant increase in cellular degeneration and fibrosis. No significant differences were observed between the positive control and the HA+AFB_1_ treatment. The histopathology studies provide further support for the findings, demonstrating that the liver of turkeys from the AFB_1_ treatment exhibited significant cellular degeneration and fat deposition. The observed enlargement and weight increase of the liver can be attributed to an accumulation of lipid content, leading to a condition known as a friable and fatty liver in the liver of chickens affected by aflatoxicosis (45). Moreover, in aflatoxicosis, the presence of severe inflammatory cell infiltrates, primarily composed of lymphocytes and heterophils, is frequently observed. This inflammatory response is believed to be a mechanism by which the body responds to the degeneration and vacuolation of hepatocytes caused by AFB_1_ exposure (46).

One of the main future challenges is to develop new procedures that may achieve comparable decontamination efficiencies in a broad spectrum of feed matrices because no general all-purpose decontamination methods could be broadly employed. Notably, the novel emerging decontamination technologies should not change the physical–chemical properties of the treated feed products significantly (47–49). The utilization of HS as feed additives has been extensively researched and has shown diverse advantages. These benefits are often observed when HS is combined with other feed additives, leading to synergistic or enhanced effects. The potential economic advantage for livestock and poultry producers lies in utilizing HA from high-quality sources and being administered according to dosage recommendations supported by research. This approach ensures the optimal use of HS and its associated benefits.

## 5. Conclusion

The results of this study provide confirmation that the presence of 250 ng AFB_1_/g had severe toxic effects on various aspects of performance, organ health, biochemical and hematological parameters, and hepatic injury in turkey poults over 28 days. The current research employed HA as a dietary inclusion in relatively small amounts, demonstrating its effective use as an additive. The findings strongly suggest that HA derived from worm compost has the potential to serve as an effective adsorbent for mitigating AFB_1_ toxicity in turkey poults. Despite these positive findings, it is important to note that research on HA as mycotoxin binders is still relatively limited, and further studies are needed to fully understand their long-term effects and optimal inclusion levels in feed formulations. Nonetheless, their promising efficacy makes them a valuable area of research for addressing mycotoxin-related challenges in poultry production. At present, our research team is conducting experiments to study the effects of HA throughout poultry production.

## Data availability statement

The original contributions presented in the study are included in the article/supplementary material, further inquiries can be directed to the corresponding authors.

## Ethics statement

The animal study was approved by all animals handing procedures complied with the Institutional Animal Care and Use Committee (IACUC) at the University of Arkansas, Fayetteville (Protocol No. 22020). The study was conducted in accordance with the local legislation and institutional requirements.

## Author contributions

JM-G: Conceptualization, Writing—review and editing, Methodology, Writing—original draft. MN-R: Conceptualization, Methodology, Writing—original draft, Investigation. SG-R: Writing—original draft, Conceptualization, Validation, Writing—review and editing, Funding acquisition, Investigation, Project administration. MÁ: Methodology, Writing—original draft, Funding acquisition, Investigation, Project administration. BS-C: Methodology, Writing—original draft. DH-P: Methodology, Writing—original draft. RM-G: Conceptualization, Software, Writing—original draft. XH-V: Writing—review and editing. JH-R: Formal analysis, Methodology, Software, Writing—review and editing. IL: Software, Writing—review and editing. RS-C: Software, Writing—review and editing. JL: Formal analysis, Writing—review and editing. AV-D: Writing—review and editing, Formal analysis, Investigation. XD: Software, Writing—review and editing. AM-A: Conceptualization, Investigation, Writing—review and editing, Validation. BH: Funding acquisition, Project administration, Writing—review and editing, Validation. GT-I: Conceptualization, Funding acquisition, Project administration, Writing—original draft, Writing—review and editing, Investigation, Validation.
