# Corrigendum: Evaluation of the efficacy of humic acids to counteract the toxic effects of aflatoxin B1 in turkey poults

**DOI:** 10.3389/fvets.2023.1346080

**Published:** 2023-12-12

**Authors:** Jesús Adonai Maguey-González, María de Jesús Nava-Ramírez, Sergio Gómez-Rosales, María de Lourdes Ángeles, Bruno Solís-Cruz, Daniel Hernández-Patlán, Rubén Merino-Guzmán, Xochitl Hernandez-Velasco, Juan Omar Hernández-Ramírez, Ileana Loeza, Roberto Senas-Cuesta, Juan D. Latorre, Alma Vázquez-Durán, Xiangwei Du, Abraham Méndez-Albores, Billy M. Hargis, Guillermo Téllez-Isaías

**Affiliations:** ^1^Posgrado en Ciencias de la Producción y de la Salud Animal, Universidad Nacional Autónoma de México (UNAM), Unidad de Posgrado, Ciudad Universitaria, Ciudad de México, México; ^2^Department of Poultry Science, University of Arkansas, Fayetteville, AR, United States; ^3^Unidad de Investigación Multidisciplinaria L14 (Alimentos, Micotoxinas, y Micotoxicosis), Facultad de Estudios Superiores (FES) Cuautitlán, UNAM, Cuautitlán Izcalli, Estado de México, México; ^4^Centro Nacional de Investigación Disciplinaria en Fisiología y Mejoramiento Animal (CENID-INIFAP), Km1 Carretera a Colon Ajuchitlán, Querétaro, México; ^5^Laboratorio 5: LEDEFAR, Unidad de Investigación Multidisciplinaria, FES Cuautitlán, UNAM, Cuautitlán Izcalli, Estado de México, México; ^6^División de Ingeniería en Nanotecnología, Universidad Politécnica del Valle de México, Tultitlan, México; ^7^Departamento de Medicina y Zootecnia de Aves, Facultad de Medicina Veterinaria y Zootecnia, UNAM, Ciudad de México, México; ^8^Veterinary Medical Diagnostic Laboratory, Department of Biomedical Sciences, University of Missouri, Columbia, MO, United States

**Keywords:** aflatoxin B1, humic acids, adsorbents, turkey poults, performance parameters

In the published article, there was an error in affiliation 8.

Instead of “Department of Veterinary Diagnostic and Production Animal Medicine, Veterinary Diagnostic Laboratory, Iowa State University, Ames, IA, United States”, it should be “Veterinary Medical Diagnostic Laboratory, Department of Biomedical Sciences, University of Missouri, Columbia, MO, United States”.

The authors apologize for this error and state that this does not change the scientific conclusions of the article in any way. The original article has been updated.

